# Effect of Pilates combined with pelvic floor muscle training on continence of post-prostatectomy incontinence in patients with different body mass index

**DOI:** 10.1186/s12894-024-01451-6

**Published:** 2024-03-28

**Authors:** Di An, Jianxia Wang, Fan Zhang, Huafang Jing, Yi Gao, Huiling Cong, Guodong Su, Miao Ye, Chunying Hu, Juan Wu, Limin Liao

**Affiliations:** 1 Department of physiotherapy 2 (PT2), China Rehabilitation Research Center the School of Rehabilitation, Capital Medical University, NO 10, Jiaomen Beilu, Fengtai district, Beijing, 100068 China; 2 Department of Intensive Care Unit, Cancer Hospital Chinese Academy of Medical Science, National Cancer Center/ National Clinical Research Center for Cancer/Cancer Hospital, Chinese Academy of Medical Sciences and Peking Union Medical College, Chaoyang District, Beijing, China; 3 Department of Urology, China Rehabilitation Research Center, Fengtai district, Beijing, China; 4 Department of physiotherapy 3(PT3), China Rehabilitation Research Center, Fengtai district, Beijing, China; 5 China Rehabilitation Research Center (CRRC), Department of Urology of Beijing Boai Hospital, Department of Urology of Capital Medical University, NO 10, Jiaomen Beilu, Beijing, 100068 China

**Keywords:** Post-prostatectomy incontinence, Pelvic floor muscle training, Pilates, BMI

## Abstract

**Background:**

Urinary incontinence symptoms severely affect older people with different body mass index (BMI).To compare the efficacy of the pelvic floor muscle training (PFMT) in patients with post-prostatectomy incontinence with different BMI.

**Methods:**

Thirty-seven patients with post-prostatectomy incontinence were included. They were divided into group A (BMI ≤ 25,12), group B (26 ≤ BMI ≤ 30,14), and group C (BMI ≥ 31,11) based on difference BMI. Three groups of patients underwent the same Pilates combined with kegel training. Participants were assessed with 1-hour pad test, the number of incontinence episodes, International Consultation on Incontinence Questionnaire and Oxford Grading Scale.

**Results:**

In the 1-hour pad test, the differences before and after training were statistically significant in all three groups of participants. Group A decreased from 81.83 ± 8.79 to 31.08 ± 5.64 g (*P* < 0.01). Group B decreased from 80.57 ± 8.87 to 35.85 ± 5.66 g (*P* < 0.01). Group C decreased from 83.55 ± 10.24 to 40.18 ± 7.01 g (*P* < 0.01). The number of incontinent episodes in group A decreased from 9.33 ± 1.07 to 3.25 ± 0.62 (*P* < 0.01). Group B decreased from 8.86 ± 1.09 to 3.79 ± 0.80 (*P* < 0.01). Group C decreased from 9.27 ± 1.10 to 4.09 ± 0.70 (*P* < 0.01). The correlation between the three groups of participants and the 1-hour pad test, with an R^2^ of 0.51. The correlation between the three groups of participants and the number of urinary incontinence episodes with a R^2^ of 0.43.

**Conclusions:**

Pelvic floor muscle training can affect the recovery of urinary continence in patients with different BMI. Maintaining a lower BMI can be beneficial for improving urinary control.

**Trial registration:**

Date of trial registration: November 27, 2023.

## Introduction

Post-prostatectomy incontinence(PPI) is an iatrogenic injury, which is difficult to avoid [1]. Its incidence typically ranges from 7 to 87%, decreasing to 5% to 20% after 1–2 years [2]. The incidence of this disease varies greatly, the main influencing factors are the amount of urine leakage during the first catheter removal, the definition of incontinence after radical prostatectomy, the selection of patients, and the surgical technique [3, 4]. To this day, the main pathophysiology of urinary incontinence is still not perfectly explained, mainly due to bladder neck dysfunction, intraoperative nerve injury and intrinsic sphincter insufficiency resulting in from sphincter relaxation [5]. The impact of urinary incontinence on society and men's daily life is enormous [6, 7], it causes a large amount of expenditure of health resources, affects the social interaction of men, and easily causes the social isolation of the elderly, it is an important indicator for the recruitment of nursing homes [8]. It increases the risk of falls [9], has an impact on the activities of daily living of partners [10], and increases mortality in home care [11].

Regarding the non-surgical treatment of urinary incontinence, it is emphasized in the guidelines of the European Urological Association and the 6th International Consultation on Urinary Incontinence that pelvic floor training should be included in the first-line treatment [12, 13]. However, Goode et al. suggest that traditional pelvic floor training is suboptimal because some patients are unwilling to undergo it [14]. In recent years, Pilates training has achieved good results in the treatment of patients with urinary incontinence after prostate surgery and can well arouse the enthusiasm of patients, it seems that Pilates training can be used in the conservative treatment of Post-prostatectomy incontinence [15, 16].

Obesity accounts for 300,000 deaths each year in the United States, and obese persons have increased all-cause mortality relative to normal-weight persons [17, 18]. Obesity also affects the urinary system. For every 5-unit increase in BMI, the risk of urinary incontinence increases by 60% to 80% [19]. In women, weight loss of more than 5% is associated with a 50% reduction in the frequency of urinary incontinence [20]. During the six months of weight loss, the incidence of all types of incontinence decreased and the number of incontinence episodes decreased, it seems that there is a benefit to the recovery of urinary incontinence with decreasing BMI [21]. However, the influence of different BMI groups on the effect of pelvic floor training in men is rarely reported. Therefore, the main objective of this paper was to determine the effects of Pilates combined with Kegel training on urinary control and pelvic floor muscle strength in patients with urinary incontinence after prostate surgery with different BMI. This is also the novelty of this paper, that is, through different BMI groups, to determine whether the effect of pelvic floor training is affected.

## Methods

### Trial design

This study was a prospective cohort controlled trial conducted at the China Rehabilitation Research Center. From January 2023 to June 2023, Registered patients experiencing PPI were recruited from both outpatient and ward settings. They were all patients who had been diagnosed with PPI, either as outpatients or in the hospital. The study was approved by the ethics committee (2023–041-01), and it was conducted independently and thoroughly. Additionally, all patients were informed of the treatment process, they all signed a consent form for rehabilitation.

### Patients

Inclusion criteria: Participants aged 60 to 80 years and within 1 year of surgery. Retropubic radical prostatectomy and laparoscopic radical prostatectomy. The patient's condition was stable and the wound healed well after surgery. 1 h pad test greater than 2 g [22]. Exclusion criteria were participants who withdrew or had incomplete data, had a history of other urologic procedures, had a history of neurologic disease, or had psychiatric symptoms. Initially, we planned to enroll 60 participants, 20 in each trial group, G*power software was used to calculate the sample size,to account for an alpha of 0.05, 80% power, and a between-group effect size of 60% [23]. A total of 49 patients were selected to participate in the study. Considering the different BMI [BMI = Height(m)/weight (kg)^2^] of the participants could affect the urinary control treatment of pelvic floor exercise [23]. We divided the patients into three groups according to the BMI range of the participants, there were 17 participants each in groups A (BMI ≤ 25) and B(26 ≤ BMI ≤ 30), and 15 participants in group C(BMI ≥ 31).

### Procedure

Through the model of group discussion, we developed a pelvic floor training program. Before the training, the pelvic floor anatomy and related knowledge were introduced to all patients, and the intake of caffeine-containing beverages was reduced. The treatment protocol was identical for all three groups of patients, who were all instructed in pelvic floor training by the same therapist. The training program was conducted by Pilates combined with pelvic floor muscle training. Pilates training we refer to studies developed by others [24, 25] (Table 1). The pelvic floor muscle training method was formulated according to other studies, each time the anus was contracted for 5 s, and the relaxation was 3 s. Our patients were encouraged to contract the anus as much as possible, with each contraction ensuring movement of the penis or scrotum. We let our patients experience which muscles force when flow is interrupted during voiding, and we do our best to strengthen the voluntary contraction of these muscles [26]. the PERFECT evaluation model was used for training assess [27]. All patients were required to train daily in the hospital, each training session lasted 45 min, and training lasted for 2 months. Urinary continence was evaluated every Saturday and Sunday, and data were recorded. Data was collected by nurses who were unaware of the study grouping and training methods.

**Table 1:** Pilates training program

Name of exercise	Description	week
1. Pilates breathing(bed)	Inhale slowly and deeply, notice the diaphragm coming down, exhale slowly and draw in the abdomen	1–2
2. Pelvic clock(bed)	Elevate pelvis toward sky	1–2
3. Only bridge(bed)	Bend knees and feet in parallel, elevate pelvis	1–2
4. Rolling like a ball(bed)	Bend hip joints and knees, hands behind thighs, roll forward and backward	1–2
5. Heel slide (bed)	Bend knees, stretch one side leg and then other leg, alternate	1–2
Homework	1 + 2 + 3 + 4 + 5	1–2
6. Pilates breathing plus	Pilates breathing plus exercise upper limbs	3–4
7. Pelvic clock plus	Pelvic clock plus exercise upper limbs	3–4
8. Bridge plus	Bridge plus exercise upper limbs	3–4
9. Heel slide plus	Heel slide plus exercise upper limbs	3–4
Homework	6 + 7 + 8 + 9	3–4
10. Foot work(chair)	Bend knees, legs parallel, bend and stretch hip joints	5–6
11. Leg alternately (chair)	Bend knees, one leg bend and other stretch, alternate	5–6
12. Side bridge (chair)	One leg on the chair, other leg on the mat, adductor press chair	5–6
13. Bridge with a chair	Bridge plus with a chair	5–6
Homework	10 + 11 + 12 + 13	
14. Lateral buckling (standing)	Lateral buckling our trunk plus exercise upper limbs	7–8
15. Rotation trunk (standing)	Rotation trunk plus exercise upper limbs	7–8
16. Diagonal buckling	Left hand touch right bend thigh, alternate	7–8
17. assist squats	Squats with our trunk stabilization	7–8
18. resisted squats	Assist squats plus resistance	7–8
Homework	14 + 15 + 16 + 17 + 18	7–8

20 times one group, and did our best to inspire our patients

### Evaluations

The primary outcome was the 1-h pad test. The patient was asked to wear the urine pad, Participants drank as much as 500 ml of water as possible and perform activities to stimulate urine leakage, such as going up and down stairs and walking, after 1 h, the urine pad was removed and weighed, and the urine leakage was judged by calculating the weight difference of the urine pad [28]. Data from 1-h pad tests were averaged over two consecutive days. According to the difference in pad weight, the patients were divided into: no incontinence < 2 g(no UI); mild urinary incontinence 2–9.9 g(mild UI); moderate UI 10–49.9 g(moderate UI) and severe UI > 50 g (severe UI) [28]. Secondary outcomes were number of incontinent episodes [26], The modified Oxford Grading Scale for recovery of pelvic floor muscle strength [29], and International Consultation Incontinence Questionnaire (ICIQ-SF) for subjective participant recovery [30]. The data were collected at the end of every week, and the changes of the evaluation indexes were recorded by the curve graph.

### Statistics

SPSS 20 software was used for statistical analysis. The basic information of the patients was analyzed by one-way ANOVA test and Fisher's exact test. The 1-h pad test and the number of incontinence episodes were expressed as means and standard deviations, and the differences between groups were determined by one-way ANOVA, and the differences among the three groups and before, during and after treatment within the group were determined by 3 × 3 binary ANOVA. The Student–Newman–Keuls [S–N-K (S)] test was used for post hoc analysis of variance. The Oxford Rating Scale and the International Incontinence Consultation Questionnaire were expressed as medians and quartiles, and the Mann–Whitney rank sum test was used to determine differences between groups, and the signed rank sum test was used to determine differences before and after treatment. Spearman rank correlation was used to determine the correlation between different BMI groups and 1-h pad test, incontinence episodes, ICIQ-SF and Oxford Rating scale results after 8 weeks of treatment. *P* < 0.05 was considered statistically significant.

## Results

**Fig. 1:**
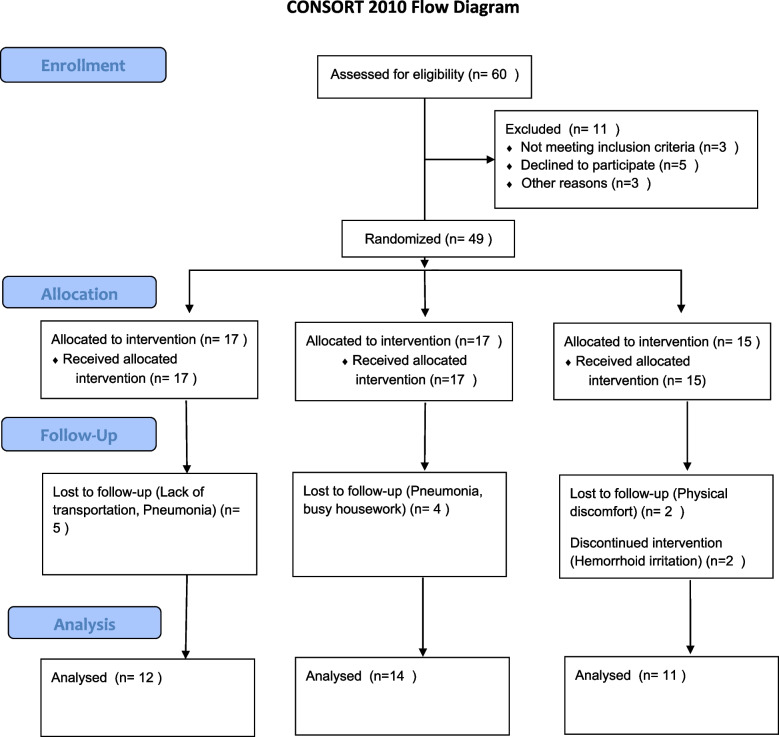
Flow chart of the study

There were no significant differences among the three groups in the basic demographic and clinical characteristics of the participants (Table 2). Of the planned 60 participants, 3 (5%) did not meet the inclusion criteria, 5 (8%) withdrew from the study, and 3 were excluded for other reasons. Subsequently, 49 participants (82%) entered the study. Finally, group A (12,20%), group B (14,23%) and group C (11,18%) completed the study (Fig. 1).

**Table 2:** The basic demographic and clinical characteristics of the 3 groups of samples

Variables	Group A	Group B	Group C	*P* value
Age (years)	74.42 ± 6.17	72.64 ± 5.92	74.91 ± 5.05	0.58
Height (m)	1.73 ± 0.05	1.73 ± 0.06	1.71 ± 0.06	0.81
The operation time	4.42 ± 1.22	4.46 ± 1.37	4.23 ± 1.23	0.88
Whether have diabetes or hypertension				0.24
Yes	7	9	9	
No	5	5	2	
Time between the surgery and the start of exercise				0.53
less than 3 months	3	2	2	
4–6 months	6	7	5	
more than 7 months	3	5	4	
Type of surgery				0.31
Radical retropubic prostatectomy	8	9	5	
Laparoscopc radical prostatectomy	4	5	6	
Whether the nerve bundle is retained				0.88
Yes	8	9	7	
No	4	5	4	
Gleason score				0.53
≤ 7	6	8	4	
> 7	6	6	7

**Fig. 2:**
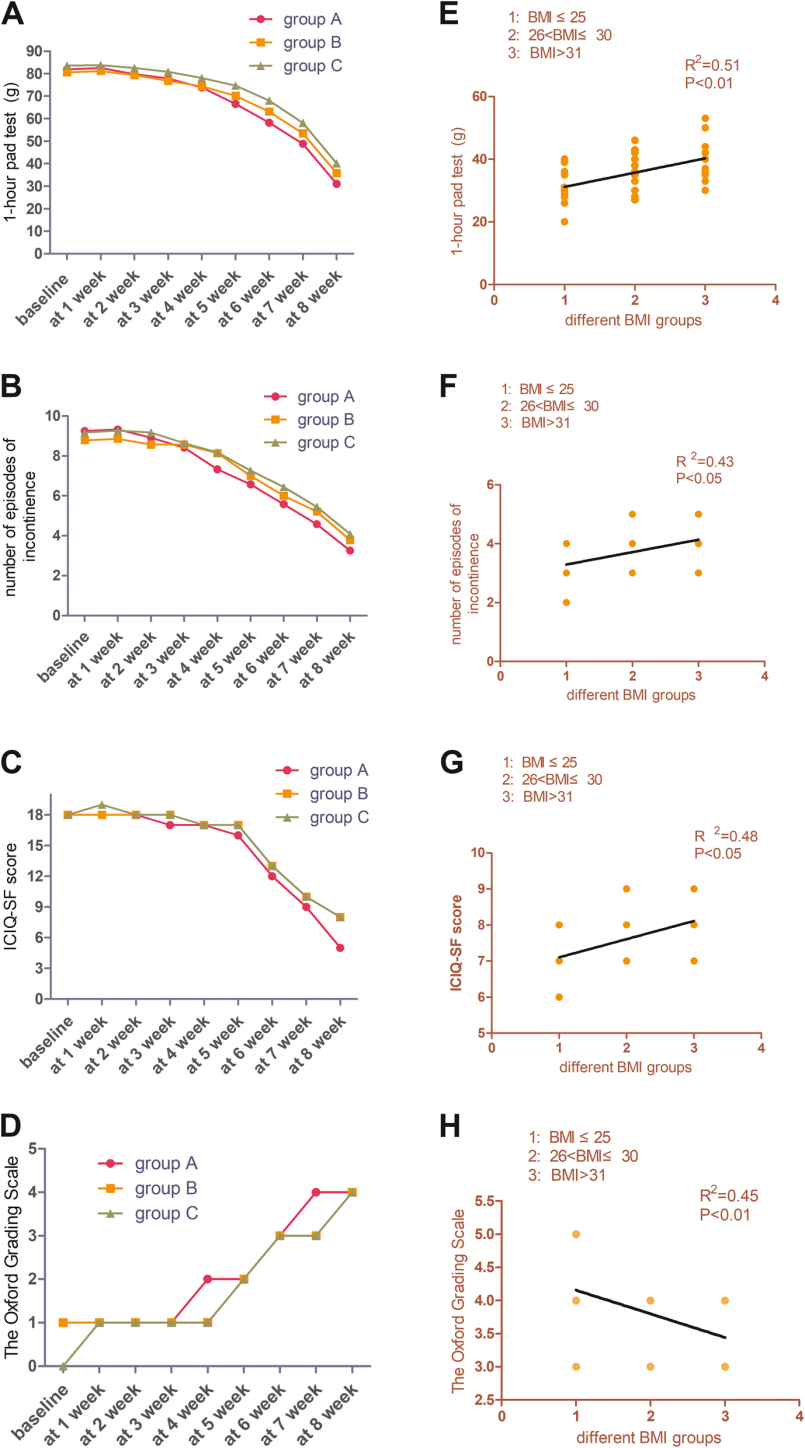
Time curves and associated outcomes for the three groups of participants

Table 3 and Fig. 2(A-D) record the differences in the evaluation indicators before, during and after treatment among the three groups of participants. In the 1-h pad test, the differences before and after treatment were statistically significant in all three groups of participants. Group A decreased from 81.83 ± 8.79 to 31.08 ± 5.64 (*P* < 0.01). Group B decreased from 80.57 ± 8.87 to 35.85 ± 5.66 (*P* < 0.01). Group C decreased from 83.55 ± 10.24 to 40.18 ± 7.01 (*P* < 0.01). The number of urinary incontinent episodes in group A decreased from 9.33 ± 1.07 to 3.25 ± 0.62 (*P* < 0.01). Group B decreased from 8.86 ± 1.09 to 3.79 ± 0.80 (*P* < 0.01). Group C decreased from 9.27 ± 1.10 to 4.09 ± 0.70 (*P* < 0.01). On the ICIQ-SF scale, group A decreased from 18(18,19) to 7(6,8) (*P* < 0.01). Group B decreased from 18(18,19) to 8(7,8) (*P* < 0.01). Group C decreased from 18(18,19) to 8(8,9) (*P* < 0.01). Group A increased from 1(0,1) to 4(4,5) on the Oxford rating scale (*P* < 0.01). Group B increased from 1(0,1) to 4(3,4) (*P* < 0.01). Group C increased from 0(0,1) to 3(3,4) (*P* < 0.01). The curve showed that there were statistical differences among the three groups after the fifth week of treatment, and the differences before and after treatment increased. Pelvic floor muscle strength close to grade 4 is beneficial to the recovery of urinary control in patients with PPI.

**Table 3:** The 3 groups were statistically compared between and within groups

Variables	Group	Baseline	At 4 week	At 8 week	*P* value
1-h pad test (Mean, standard deviation	A	81.83 ± 8.79	73.83 ± 7.26	31.08 ± 5.64	< 0.01
	B	80.57 ± 8.87	74.35 ± 6.52	35.85 ± 5.66	< 0.01
	C	83.55 ± 10.24	78.09 ± 8.60	40.18 ± 7.01	< 0.01
*P* value		0.73	0.34	< 0.01	
Incontinence episode frequency (Mean, standard deviation)	A	9.33 ± 1.07	7.33 ± 0.65	3.25 ± 0.62	< 0.01
	B	8.86 ± 1.09	8.14 ± 0.95	3.79 ± 0.80	< 0.01
	C	9.27 ± 1.10	8.18 ± 0.60	4.09 ± 0.70	< 0.01
*P* value		0.48	< 0.05	< 0.05	
ICIQ-SF score (median, interquartile range)	A	18(18,19)	17(16,17)	7(6,8)	< 0.01
	B	18(18,19)	17(17,17)	8(7,8)	< 0.01
	C	19(18,19)	18(17,18)	8(8,9)	< 0.01
*P* value		0.51	0.06	< 0.05	
Oxford Grading Scale (median ,interquartile range)	A	1(0,1)	1(2,2)	4(4,5)	< 0.01
	B	1(0,1)	1(1,2)	4(3,4)	< 0.01
	C	0(0,1)	1(1,2)	3(3,4)	< 0.01
*P* value		0.35	< 0.05	< 0.05

In the 1-h pad test, there was no significant difference among the three groups before treatment (*P* = 0.73), but there was a significant difference after treatment (*P* < 0.05). Post hoc test showed that only group A and group C had significant differences (*P* < 0.01). In the number of urinary incontinent episodes, there was no significant difference among the three groups before treatment (*P* = 0.48), but there was significant difference after treatment (*P* < 0.05). Post hoc test showed that only group A and group C had significant differences (*P* < 0.01). In ICIQ-SF scores, there was no significant difference among the three groups before treatment (*P* = 0.51), but there was significant difference after treatment (*P* < 0.05). Post hoc test showed that only group A and group C had significant differences (*P* < 0.01). In the Oxford rating scale, there was no significant difference among the three groups before treatment (*P* = 0.35), but there was significant difference after treatment (*P* < 0.05). Post hoc test showed that only group A and group C had significant differences (*P* < 0.01).

Figure 2 (E–H) shows the correlations with the four evaluation outcomes after 8 weeks of treatment for the three groups of participants. Panel E shows the correlation between the three groups of participants and the 1-h pad test, with an R^2^ of 0.51. Panel F shows the correlation between the three groups of participants and the number of urinary incontinence episodes, with a R^2^ of 0.43. Panel G shows the correlation between the three groups of participants and the ICIQ-SF, with a R^2^ of 0.48, and panel H shows the correlation between the three groups of participants and the Oxford rating scale, with a R^2^ of 0.45.

## Discussion

### Effects of different BMI on patients with PPI

Obese people have increased abdominal pressure, which will affect the pressure of abdominal organs, especially the change of bladder pressure. Subak et al. found a significant effect of weight change on urodynamic outcomes, Weight loss can reduce initial intravesical pressure and intravesical pressure at maximum capacity, improvement in Valsalva leak point pressure and change in bladder pressure after weight loss were independent predictors of improvement in urinary incontinence [23]. This confirmed our finding that there were differences among the 3 groups and that group A had a faster recovery in pelvic floor muscle strength and Incontinence episode frequency at week 4. There are differences in initial bladder pressure and detrusor instability due to abdominal pressure in patients with post-prostatectomy incontinence with different BMI [19, 23]. In addition, the risk of urinary incontinence is elevated following surgical resection of the prostate and surrounding tissues, particularly the urethral sphincter complex [31]. This results in obese patients with post-prostatectomy incontinence being more likely to develop incontinence symptoms. However, the aggravation of urinary incontinence symptoms will increase the risk of falls and aggravate the work of future nursing and family care [32]. In the trial, we found statistical differences between groups A and C only in the 1-h pad test. We speculate that controlling BMI in group B level by reasonable weight loss in patients with urinary incontinence with BMI > 31 can greatly improve the urinary control ability of patients with post-prostatectomy incontinence. We suggest that patients with post-prostatectomy incontinence should have a reasonable diet and weight control at the same time of pelvic floor training, which can reduce the difficulty of home care.

### Advantages of pilates training combined with Kegel training

Some previous studies have confirmed that Pilates combined with Kegel training can effectively improve urinary control in patients with post-prostatectomy incontinence [24]. By transferring the traditional Kegel training for trunk stability to the Kegel training for trunk instability, the recruitment rate of trunk core muscles can be stimulated by different postural transitions [33], the study conducted by Stafford et al. revealed that the activation of core muscle groups can effectively enhance pelvic floor muscle contraction [34], abdominal muscle activity can improve pelvic floor muscle strength [35], so as to better promote the improvement of pelvic floor muscle strength [15, 16]. In trials, Pilates training mostly involved different positions. The shortcomings of traditional pelvic floor training can be further optimized by combining Kegel training with different postural changes. In addition, Dias et al. found that Pilates training can better promote the enthusiasm of patients for pelvic floor training compared with traditional pelvic floor training [36]. Therefore, we believe that Pilates combined with Kegel training is one of the best ways to perform pelvic floor training for patients with urinary incontinence.

### Limitation

There was no blind treatment among the three groups, which may affect the training enthusiasm of different groups. However, we provided patient guidance to patients in each group during training to maximize the enthusiasm of patients, training methods were identical in all three groups to maximize the intention-to-treat analysis. Long-term follow-up is lacking in our study, we will continue to explore the long-term effects of pelvic floor exercise in patients with different BMI in future studies. Although none of the participants in the three groups had received professional pelvic floor training before entering the study, most of them obtained more or less relevant information about pelvic floor muscle training through doctor consultation, Internet and other ways. Therefore, our study is not fully representative of pelvic floor muscle training results in all populations with different BMI. The benefits of pelvic floor muscle training often diminish and disappear as patients discontinue the program, while simultaneously focusing too much on training intensity, which may potentially impact patient recovery in the long run. Therefore, future studies will aim to refine the training protocol.

## Conclusion

Different BMI groups can affect the recovery of urinary control after pelvic floor muscle training. It is recommended that patients should control body weight reasonably while performing pelvic floor training. Pilates combined with Kegel training can promote the recovery of urinary control and pelvic floor muscle strength in patients with urinary incontinence after prostate surgery.

## Data Availability

Our Data is provided within the manuscript or supplementary information files.
